# Clinical Implications of Remote Dielectric Sensing-Guided Management

**DOI:** 10.3390/jcm13102906

**Published:** 2024-05-14

**Authors:** Yu Nomoto, Teruhiko Imamura, Toshihide Izumida, Nikhil Narang, Koichiro Kinugawa

**Affiliations:** 1 Second Department of Internal Medicine, University of Toyama, Toyama 930-0194, Japan; 2 Advocate Christ Medical Center, Oak Lawn, IL 60453, USA

**Keywords:** heart failure, congestion, diuretics

## Abstract

**Background:** Remote dielectric sensing (ReDS) systems can quantify the degree of pulmonary congestion rapidly and non-invasively. However, the clinical implications of ReDS-guided medication adjustment remain uncertain. **Methods:** Patients hospitalized to treat cardiovascular diseases, including heart failure, valvular disease, and coronary artery disease, and underwent ReDS measurement before index discharge between 2021 and 2022 were included. According to our institutional protocol, ReDS values were blinded to the attending clinicians until February 2022 (blind period). After the period, ReDS values were timely opened to the attending clinicians, and medications such as diuretics were adjusted according to the ReDS values (target value between 20% and 35%) before index discharge (open period). A composite primary outcome of all-cause death and heart failure readmissions was compared between the two groups. **Results:** A total of 183 patients were included (median 79 years old, 101 men), consisting of 138 patients in the blind period and 45 patients in the open period. During a median of 646 (401, 818) days after the index discharge, 33 patients experienced the primary outcome of interest. Management during the open period, where medications were adjusted according to ReDS values, was independently associated with a lower incidence of the primary outcome with an adjusted hazard ratio of 0.22 (95% confidence interval 0.05–0.94, *p* = 0.041), as compared with those of the blind period. **Conclusions:** According to the findings of the present retrospective study, ReDS-guided management may have the potential to reduce the risk of mortality and heart failure admission in individuals hospitalized for cardiovascular diagnoses. Further prospective randomized control trials involving those with a variety of background etiologies and clinical scenarios are warranted to validate our findings and establish optimal ReDS-guided management.

## 1. Introduction

Subclinical levels of pulmonary congestion may manifest as a harbinger of deleterious clinical consequences in individuals living with chronic heart failure [1]. Consequently, the management and treatment of pulmonary congestion are important therapeutic targets before discharge in patients presenting with acute-on-chronic worsening heart failure, for without such interventions portends a high risk of readmission and inevitably worse clinical outcomes [2].

Nevertheless, this imperative is not without its challenges, stemming from the absence of tools with adequate precision required to quantify the extent of pulmonary congestion [3]. While right heart catheterization stands as the gold standard for this purpose, its intrinsic invasiveness precludes its routine application in all patients with suspected decompensated heart failure [4]. As an alternative, non-invasive modalities such as chest X-ray, plasma B-type natriuretic peptide, lung ultrasound, and physical examination are acceptable though imprecise alternatives to quantify clinical congestion [5].

These modalities are widely applied to assess pulmonary congestion in daily clinical practice. However, these are qualitative and require expert techniques for reading. This is problematic not only for individuals with heart failure but also for those without an obvious diagnosis of heart failure, who also may have subclinical pulmonary congestion that results in worse clinical outcomes.

A recent technological innovation, the remote dielectric sensing (ReDS) system, has emerged as a non-invasive solution for estimating lung fluid volume [6]. Functioning based on electromagnetic energy, this technology provides a percentage representation of lung fluid volume on a monitor within a minute. The capacity of ReDS systems to quantify the degree of pulmonary congestion has been rigorously validated through comparative analyses with established modalities, including computed tomography, right heart catheterization, chest X-ray, lung ultrasound, and plasma B-type natriuretic peptide levels [7]. Noteworthy is the approval of ReDS by regulatory bodies including the FDA and CE for monitoring lung fluid levels, extending to countries including Japan. However, the clinical implication of ReDS-guided management has not been well demonstrated in real-world clinical practice.

In our institutional framework, ReDS values were judiciously ascertained in a blinded fashion before the index discharge per established protocols between August 2021 and February 2022. Subsequently from February 2022, attending clinicians were apprised of the ReDS values, guiding the titration of doses of medications such as diuretics in alignment with institutional protocols. We performed a comparative assessment of post-discharge clinical outcomes between the ReDS-guided management cohort (open period group) and the conventionally treated group without ReDS values (blind period group) in patients admitted with cardiovascular conditions.

## 2. Methods

### 2.1. Study Design

At our institution, a paradigm shift in therapeutic strategies using ReDS measurements was instituted as of February 2022. Preceding this time frame, ReDS values were ascertained in a blinded fashion, with the subsequent management of patients’ congestion through standard medication protocols, unguided by ReDS values (blind period group).

Commencing from February 2022, an updated therapeutic approach was introduced, wherein patient management before the index discharge involved attention to ReDS values, as delineated below (open period group).

This retrospective study aimed to evaluate the prognostic ramifications of ReDS guidance by comparing rates of a combined primary outcome, encompassing all-cause mortality and heart failure admissions, between the two cohorts (blind period group versus open period group).

### 2.2. Patient Selection

Individuals hospitalized within our institutional wards for cardiovascular diseases, including heart failure, valvular disease, and coronary artery disease, routinely underwent ReDS measurements as a standard protocol preceding the index discharge.

Patients exhibiting incongruent physiological parameters, such as a body mass index below 15 kg/m^2^ or exceeding 30 kg/m^2^, body height less than 140 cm, and those with severe scoliosis, were excluded from ReDS measurements, given its technical limitations [7]. Similarly, patients with active pulmonary disorders, including pneumonia, chronic obstructive pulmonary disease, severe pulmonary effusion, and lung cancer, were ineligible for ReDS assessments and were excluded from this study after their medical visit, given their impacts on the ReDS values [7]. Additionally, individuals unable to maintain a seated position or tolerate the application of ReDS devices for one minute were excluded from ReDS measurements, given the technical challenge. Notably, patients who died during the index hospitalization were excluded from the study cohort because of the lack of their ReDS values.

Written informed consent was obtained from all enrolled patients upon admission. The protocol received ethical approval from the local ethics committee (MTK2020007, 3 March 2021).

### 2.3. ReDS Measurements

**Figure 1:**
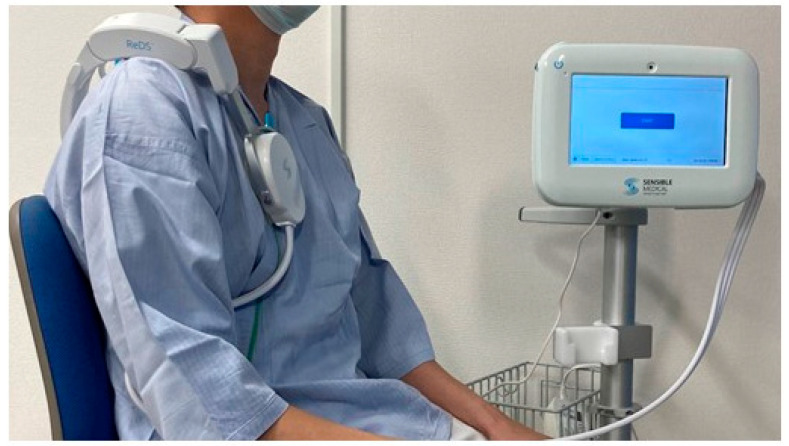
ReDS system. A patient is asked to sit on a chair with a back seat under natural breathing. The patient wears a ReDS sensor and waits for approximately 60 s. The ReDS value, a representative of the percentage of lung fluid amount, is displayed on the screen.

The ReDS system constitutes a recent advancement in non-invasive technology, relying on electromagnetic energy for the expeditious quantification of lung fluid levels within a minute (Figure 1) [6]. The device can be applied directly on the skin. However, patients are generally asked to wear underwear during ReDS measurements.

This innovative system operates through the emission of low-power electromagnetic signals between two sensors seamlessly integrated into a wearable device. The scrutinized signal encapsulates the dielectric properties of the lung segment situated between these sensors. Given the significantly disparate dielectric coefficients of water and air—wherein water exhibits a markedly high dielectric coefficient and air a low dielectric constant—the tissue’s dielectric coefficient is primarily governed by its fluid content. Consequently, the volumetric proportion of lung fluid (expressed as the ratio of lung fluid volume to total volume) can be meticulously computed.

ReDS values were obtained within a time frame of 45 to 60 s, with patients assuming a seated and resting position while breathing normally without any breath holding. The ReDS device, comprised of two sonars, was positioned on the right shoulder. Subsequently, the calculated ReDS values were promptly displayed on the monitor, presented as a percentage denoting lung fluid volume relative to the total lung volume.

### 2.4. Management of Pulmonary Congestion

Within the blind period group, the handling of pulmonary congestion adhered to standardized protocols involving the administration of medications such as diuretics. This approach relied on the evaluation of physical examination, laboratory data, chest X-ray results, and, when applicable, echocardiography. Although ReDS values were recorded for this cohort, these findings remained concealed from attending clinicians and were solely used for research purposes by non-attending researchers.

**Figure 2:**
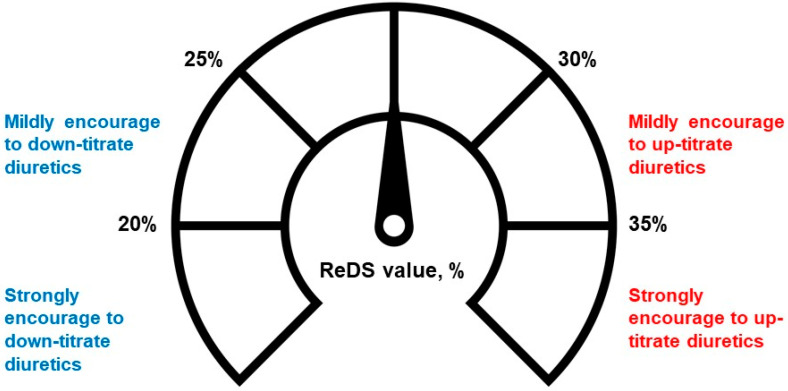
Institutional protocol to adjust diuretics according to ReDS value. A manufacturer proposes a normal range of ReDS value between 20% and 35%. As per institutional protocol, we adjusted the dose of diuretics according to ReDS values to maintain appropriate lung fluid amount during the open period, during which ReDS values were open to attending clinicians for guidance. We encouraged the adjustment of diuretics when ReDS values were between 30% and 35% and highly recommended the up-titration in cases of ReDS values >35%. We encouraged the slight down-titration of the diuretics dose when ReDS values were between 20% and 25% and highly recommended the down-titration of the diuretics dose in cases of ReDS values <20%.

In contrast, the open period group underwent a distinct management algorithm: ReDS-guided management. ReDS values were diligently obtained before the index discharge, and these values played a pivotal role in guiding the adjustment of medications, particularly diuretic dosages, by referencing the previous literature (Figure 2) [8,9]. Specifically, if the ReDS values surpassed the 35% threshold, an up-titration of diuretic doses was forced to be implemented, in principle. If the ReDS values were between 30% and 35%, an up-titration of diuretics doses was recommended but not mandatory. The doses of other medications were also adjusted at the discretion of the attending clinicians.

Conversely, if the ReDS values fell below 20%, a down-titration of diuretic doses was ordered by the treatment team. If the ReDS value was between 20% and 25%, a down-titration of diuretic doses was encouraged, but not considered mandatory. Similarly, doses of other medications were also adjusted at the discretion of the attending clinicians. Ancillary nutrition services were offered to hospitalized patients with cardiovascular conditions.

### 2.5. Post-Discharge Management

All patients were followed at our institutional clinic or affiliated centers by board-certified cardiologists in a standard manner with one visit per month, in principle. In addition to the physical examination and vital sign measurement, chest X-ray, electrocardiogram, transthoracic echocardiography, or any other examinations were followed, if applicable. Medications were adjusted at the discretion of the attending clinicians.

### 2.6. Data Collection

Demographics, comorbidity, laboratory, echocardiographic, and medication data obtained before index discharge were retrieved from the medical chart as baseline characteristics, together with ReDS values. Data regarding changes in heart failure medication (initiation/termination) were obtained. Of note, dose change (dose up/down) data were retrieved for loop diuretics.

The time of index discharge was defined as day 0. After index discharge, all-cause death and heart failure admission were counted as primary outcomes. The events were adjudicated by two independent researchers: YN and TI (Izumida).

### 2.7. Statistical Analysis

Continuous variables were expressed as medians and interquartile ranges (IQR) and compared between the two groups using the Mann–Whitney U test. The normality of their distribution was confirmed using the Shapiro–Wilk test. Categorical variables were expressed as numbers and percentages and compared between the two groups using the chi-square test or Fischer’s exact test.

Patients were divided into two groups based on the concept of ReDS value management: the blind period group and the open period group. In the blind period group, ReDS values were obtained but blinded to the attending physicians. In the open period group, ReDS values were also obtained and evaluated timely by the attending physicians. Diuretic therapy was adjusted according to the ReDS values as detailed above.

The impact of different management practices on the composite primary outcomes of all-cause death and heart failure admission was evaluated using Cox proportional hazard ratio regression analysis. Eleven potential variables that may have significant prognostic impacts were selected as potential confounders of the independent variable (i.e., open period versus blind period) and included in the univariable analysis. Variables with *p* < 0.05 in the univariable analysis were included in the multivariable analysis with a forced method. A Kaplan–Meier analysis and a log-rank test were applied to compare the cumulative incidence of the primary outcomes between the groups.

## 3. Results

### 3.1. Baseline Characteristics

A total of 183 patients were included in this retrospective study (Table 1). All patients were hospitalized for cardiovascular diseases and underwent ReDS measurements at the index discharge. The median age was 79 (72, 84) years, and 101 (55%) patients were men. The median body mass index was 21.4 (19.6, 25.3) kg/m^2^. Of the admitted patients, 112 had heart failure, 50 had coronary artery disease, and 87 had valvular disease. No patients had obvious pulmonary diseases such as pulmonary pneumonia, chronic obstructive pulmonary disease, and lung cancer. Serum creatinine was 1.1 (0.9, 1.3) mg/dL and plasma B-type natriuretic peptide was 127 (80, 254) pg/mL. Ninety-three patients (51%) received diuretics.

### 3.2. ReDS Value

**Figure 3:**
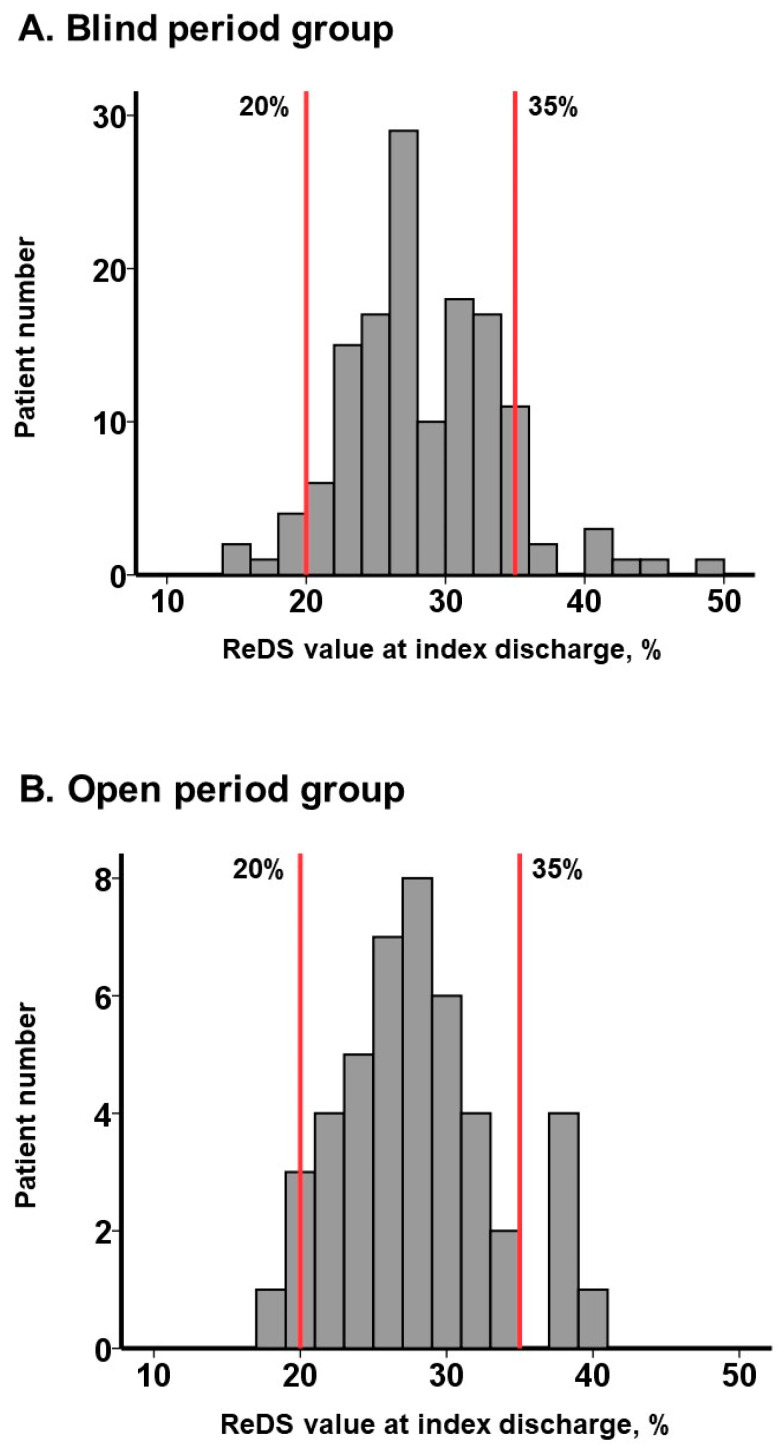
Distribution of ReDS values at index discharge in the blind period (**A**) and in the open period (**B**). During the blind period, ReDS values were measured but were blinded to attending clinicians. The data were reviewed retrospectively after the termination of the observational period by the independent researchers. During the open period, ReDS values were measured and opened to the attending clinicians for the guidance of diuretics dose adjustment. A manufacturer recommends a normal range of ReDS values between 20% and 35% (red lines). ReDS values outside of this normal range were assumed as abnormal.

The median ReDS value in the whole cohort was 27% (24%, 31%). The distribution of ReDS values in the blind period and the open period is displayed in Figure 3A,B. Their distribution did not significantly differ between the two groups (*p* = 0.62). In the retrospective review, 9 patients had ReDS values <20% and 14 patients had ReDS values >35% in the blind period group (*N* = 138; Figure 3A). In the open period group (*N* = 45), four patients had ReDS values <20% and five patients had ReDS values >35% (Figure 3B). Of note, in the open period group, the dose of diuretics was forced to be adjusted in individuals with abnormal ReDS values (i.e., <20% or >35%).

### 3.3. Medication Change

Medication changes during index hospitalization were summarized in Table 2. Medications were initiated or terminated during the index hospitalization. Heart failure medications tended to be adjusted more in the open group. Of note, the dose of loop diuretics was significantly adjusted at a higher rate in the open group (*p* < 0.001).

### 3.4. Prognostic Impact of ReDS-Guided Management

During a median observation period of 646 (401, 818) days after the index discharge, there were 12 deaths and 23 heart failure admissions, representing the combined primary outcome.

Among 11 potential confounders, three variables were significantly associated with the primary outcome, including lower left ventricular ejection fraction, the use of loop diuretics, and the open period (i.e., ReDS-guided management) (*p* < 0.05 for all; Table 3). Among them, the open period (versus the blind period) was independently associated with the primary outcome with an adjusted hazard ratio of 0.22 (95% confidence interval 0.05–0.94, *p* = 0.041).

### 3.5. Stratification of Clinical Outcome

**Figure 4:**
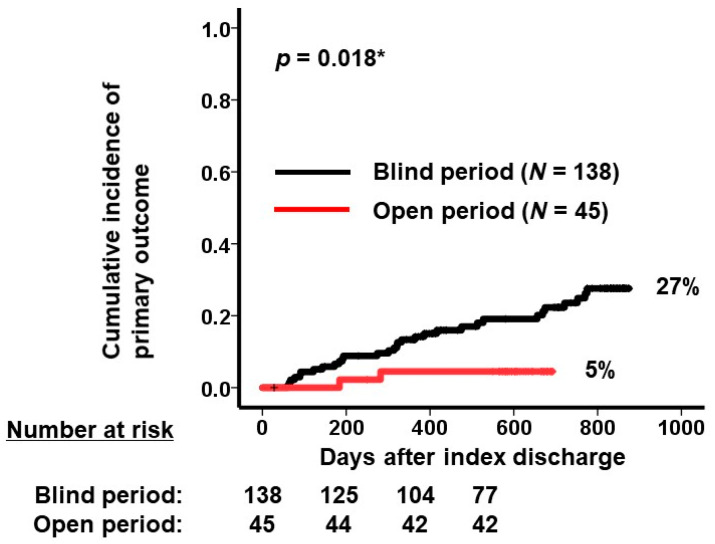
Cumulative incidence of primary outcome in the blind period (black curve, *N* = 138) and the open period (red curve, *N* = 45). The primary outcome consisted of all-cause death and heart failure admission after index discharge. * *p* < 0.05 by log-rank test.

During the observational period after index discharge, the open period group (i.e., ReDS-guided group) had a significantly lower cumulative incidence of the primary outcome than the blind period group (5% vs. 27%, *p* = 0.018; Figure 4). In detail, 2/45 patients in the open period group and 21/138 patients in the blind period group had heart failure hospitalization (*p* = 0.058). Mortality was encountered in 2/45 patients in the open period group and 10/138 patients in the blind period group (*p* = 0.51).

**Figure 5:**
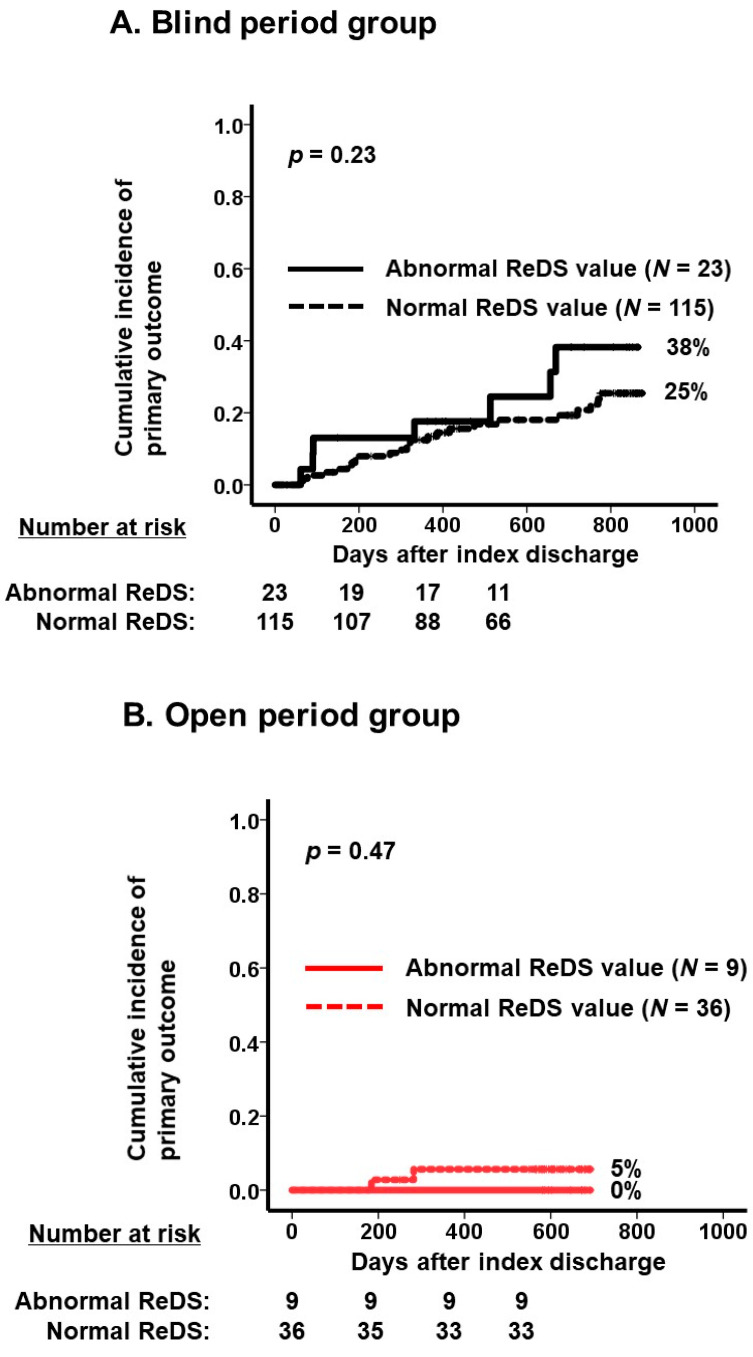
Cumulative incidence of primary outcome in each period: blind period (black curve; (**A**)) and open period (red curve; (**B**)). In each group, the cohort was divided into two groups according to the normality of ReDS values: Abnormal ReDS value group versus normal ReDS value group. An abnormal ReDS value was defined as <20% or >35% according to the manufacturer’s proposal. Two curves were compared using the log-rank test.

Among the blind period group (*N* = 138), a total of 23 patients had abnormal ReDS values (i.e., <20% or >35%). These patients tended to have a higher cumulative incidence of the primary outcome than those with normal ReDS values (38% versus 25%, *p* = 0.23; Figure 5A). Of note, these ReDS values were blinded to the attending clinicians and retrospectively reviewed by independent adjudication after the end of the observation period.

Among the open period group (*N* = 45), nine patients had abnormal ReDS values. The cumulative incidence of the primary outcome was lower regardless of the abnormality of ReDS values (*p* = 0.47, Figure 5B). In this group, medications were aggressively adjusted in individuals with abnormal ReDS values.

## 4. Discussion

In this retrospective study, we investigated the prognostic impact of ReDS-guided management in patients hospitalized for cardiovascular diseases. In the ReDS-guided management group, heart failure medications, particularly loop diuretics, were more aggressively adjusted to abnormal ReDS values. ReDS-guided management (i.e., the open period group) was independently associated with the lower cumulative incidence of the composite primary outcome consisting of all-cause death and the risk of heart failure admission. In the blind group, individuals with abnormal ReDS values (<20% or >35% according to the manufacturer’s proposal) tended to have a higher cumulative incidence of the primary outcome. On the contrary, individuals with abnormal ReDS values had a lower cumulative incidence of the primary outcomes statistically comparable to those with normal ReDS values in the open period group receiving ReDS-guided management.

### 4.1. Assessment of Pulmonary Congestion

Assessment of pulmonary congestion is an important component of the optimal management of the patient presenting with decompensated heart failure to improve clinical status and reduce the risk of readmission [10]. However, the accurate quantification of pulmonary congestion is challenging. The ReDS system is a recently innovated non-invasive modality to quantify the degree of lung fluid by utilizing electromagnetic power [6]. ReDS values have been validated to strongly correlate with those measured using computed tomography with specific applications [11].

ReDS values have a modest correlation with other tools used to evaluate clinical status, including physical examination, lung echocardiography, and chest X-ray [7]. These conventional modalities are practical and convenient but qualitative. They are useful in cases of overt congestion but are insensitive to subclinical changes that can be targeted as the key precursor to the development of clinical symptoms.

ReDS values also have a modest correlation with plasma B-type natriuretic peptide and plasma volume. ReDS values focus on the lung fluid, whereas these conventional modalities are affected by other confounders, including renal function, the presence of atrial fibrillation, and the degree of systemic congestion.

ReDS values represent lung fluid volume, whereas pulmonary artery wedge pressure, measured via invasive right heart catheterization, is representative of intracardiac pressures. Thus, they show only a moderate correlation.

Compared to these conventional modalities, the ReDS system may have an advantage in its ability to quantify lung fluid amount, non-invasiveness, no requirement of expert technique, and quick measurement [6]. Furthermore, the ReDS system may give us insights regarding hypovolemia when ReDS values are lower. Hypovolemia is also often challenging to be accurately assessed using conventional modalities.

Thus, we hypothesized that utilizing the ReDS system might further assist clinicians in adjusting medical therapies with precision in high-risk cohorts. Consistently, heart failure therapies, particularly loop diuretics, were more aggressively up-titrated in the open group under ReDS guidance.

### 4.2. Prognostic Impact of ReDS-Guiding

The presence of pulmonary congestion is associated with a significantly increased risk of cardiovascular mortality and morbidity in patients with acute-on-chronic heart failure [2]. The recent literature has shown that persistent elevations of ReDS values during index hospitalization were associated with a higher risk of all-cause death and incident heart failure hospitalization [12]. A previous retrospective study demonstrated that the post-discharge immediate measurement of ReDS values and medication adjustment was associated with a lower risk of 30-day cardiovascular admission [8]. Another retrospective study demonstrated the clinical benefit of the post-discharge 90-day daily follow-up of ReDS values in reducing heart failure readmission compared with pre-treatment and post-treatment periods [9].

The current study strengthened these findings by demonstrating the advantage of ReDS-guided medication adjustment in reducing the risk of mortality and heart failure admission rates in the follow-up year following discharge. Interestingly, individuals with abnormal ReDS values, including both higher and lower values, tended to have worse clinical outcomes in the blind period group. ReDS values were blinded to the attending clinicians in this period, and residual pulmonary congestion (or hypovolemia) may have been present and potentially led to worse clinical outcomes. Clinically obvious congestion/hypovolemia can be well managed by referencing conventional modalities alone. ReDS system may have a particular advantage in displaying subclinical congestion/hypovolemia, which is challenging to identify using conventional modalities alone.

On the contrary, individuals with abnormal ReDS values were observed to have no statistically significant difference in outcomes in the open period group. Even subclinical congestion/hypovolemia would have been identified with abnormal ReDS values and such abnormalities may have been correctly treated.

### 4.3. Limitations

The current study has several potential limitations. The sample size was moderate. This study was conducted in a single center involving individuals hospitalized for cardiovascular diseases. We did not include individuals in high-care units or those with other diseases. We also excluded those with obvious pulmonary diseases given their impacts on ReDS values. We investigated the clinical implication of ReDS-guided management by comparing it with conventional management without ReDS guidance. However, this was not a randomized control trial. Most of the baseline characteristics were not significantly different between the two groups, but we cannot deny the presence of potential confounders. This is a proof-of-concept preliminary study and further larger-scale randomized control trials are warranted to validate our findings. CardioMEMS is another tool to measure pulmonary artery pressure and help us adjust heart failure medication, although it is not available in many countries [13]. Comparative studies between ReDS and CardioMEMS remain a future concern [14].

We used a cutoff of ReDS values to consider interventions that was proposed by the manufacturer: 20% and 35%, similar to the previous literature [8,9]. However, its clinical validity has not yet been established and further studies are needed to find the optimal cutoff [15]. Particularly, the lower limit of ReDS values, which indicates hypovolemia, may require further studies to be accurately defined.

In the ReDS-guided group (open period group), medications were aggressively adjusted. However, we did not follow ReDS values. The trajectory of ReDS values during the observation period remains uncertain. The implications of the repeated adjustment of medication with ReDS guidance need further study.

We only followed major endpoints. The impact of ReDS guidance on other parameters such as plasma B-type natriuretic peptide levels, echocardiographic, and hemodynamics data along with exercise capacity remain topics for future investigation.

Other modalities that estimate thoracic congestion by utilizing impedance technology have also been introduced [16,17,18]. Although further comparison studies are warranted, the ReDS system seems to have the advantage over these conventional ones in its reproducibility, easy measurement, correlation with other modalities, and association with future clinical outcomes in a variety of clinical scenarios.

## 5. Conclusions

The ReDS-guided management of medication dosage at the time of index discharge may have the potential to reduce the risk of mortality and heart failure admission in individuals hospitalized for cardiovascular diseases by ameliorating congestion and hypovolemia, as compared with the standard therapeutic strategy utilizing conventional modalities such as chest X-ray. The ReDS system may be particularly useful to identify individuals with subclinical congestion/hypovolemia that cannot be identified using conventional modalities alone. Nevertheless, the applicability of our findings cannot simply be adopted to general cohorts given the retrospective nature of this study and potential selection bias. Further prospective randomized control trials are warranted to validate our findings and establish optimal ReDS-guided management.

## Figures and Tables

**Table 1:** Baseline characteristics.

	Total (*N* = 183)	Blind Period (*N* = 138)	Open Period (*N* = 45)	*p*-Value
Demographics				
Age, years	79 (72, 84)	78 (72, 84)	80 (77, 84)	0.12
Men	101 (55%)	75 (54%)	26 (58%)	0.41
Body mass index, kg/m^2^	21.4 (19.6, 25.3)	23.0 (20.2, 26.3)	21.3 (19.8, 27.0)	0.58
Systolic blood pressure, mmHg	105 (96, 123)	109 (97, 122)	104 (94, 123)	0.49
Comorbidity				
Hypertension	143 (78%)	109 (79%)	34 (76%)	0.38
Dyslipidemia	103 (56%)	78 (57%)	25 (56%)	0.52
Diabetes mellitus	59 (32%)	44 (32%)	15 (33%)	0.50
Atrial fibrillation	97 (53%)	73 (53%)	24 (53%)	0.55
Heart failure	112 (61%)	83 (60%)	29 (64%)	0.37
Coronary artery disease	50 (27%)	39 (28%)	11 (24%)	0.39
Valvular disease	87 (48%)	64 (46%)	23 (51%)	0.35
History of stroke	33 (18%)	21 (15%)	12 (27%)	0.068
Laboratory data				
Hemoglobin, g/dL	12.1 (11.1, 13.4)	12.1 (11.1, 13.1)	12.4 (11.0, 13.4)	0.77
Serum albumin, g/dL	3.5 (3.2, 3.8)	3.5 (3.2, 3.8)	3.6 (3.3, 3.8)	0.37
Serum sodium, mEq/L	139 (137, 140)	139 (137, 141)	139 (137, 141)	0.91
Serum creatinine, mg/dL	1.1 (0.9, 1.3)	1.0 (0.8, 1.4)	1.1 (0.9, 1.3)	0.14
Plasma B-type natriuretic peptide, pg/mL	127 (80, 254)	128 (71, 254)	127 (91, 255)	0.25
Echocardiographic data				
Left ventricular end-diastolic diameter, mm	51 (46, 57)	48 (44, 56)	49 (46, 54)	0.87
Left ventricular ejection fraction, %	54 (45, 66)	56 (46, 67)	63 (54, 69)	0.033 *
Left atrial diameter, mm	41 (36, 51)	42 (37, 50)	43 (38, 48)	0.71
Medication				
Beta-blocker	137 (75%)	104 (75%)	33 (73%)	0.46
Renin-angiotensin system inhibitor	166 (91%)	123 (89%)	43 (96%)	0.16
Mineralocorticoid receptor antagonist	79 (43%)	57 (41%)	22 (49%)	0.24
Sodium-glucose cotransporter 2 inhibitor	63 (34%)	50 (36%)	13 (29%)	0.24
Diuretics	93 (51%)	68 (49%)	25 (56%)	0.29
ReDS value, %	27 (24, 31)	27 (24, 32)	27 (24, 30)	0.62

ReDS, remote dielectric sensing. ReDS value was blinded to the attending clinicians during the blind period, whereas ReDS values were open and diuretics were adjusted according to the ReDS values during the open period. Continuous variables were stated as medians (25% interquartile, 75% interquartile) and compared between the two groups using the Mann–Whitney U test. Categorical variables were stated as numbers and percentages and compared between the two groups using a chi-square test or Fischer’s exact test. * *p* < 0.05.

**Table 2:** Changes in medication during index hospitalization.

	Blind Group (*N* = 138)	Open Group (*N* = 45)	*p*-Value
Beta-blocker			
Initiation/Termination	17/4	9/2	0.37
Any change	21 (15%)	11 (24%)	0.12
Renin-angiotensin system inhibitor			
Initiation/Termination	20/1	12/0	0.15
Any change	21 (15%)	12 (27%)	0.068
Mineralocorticoid receptor antagonist			
Initiation/Termination	19/4	12/1	0.13
Any change	23 (17%)	13 (29%)	0.061
Sodium-glucose cotransporter 2 inhibitor			
Initiation/Termination	19/3	8/2	0.56
Any change	22 (16%)	10 (22%)	0.23
Loop diuretics			
Dose-up/down	16/7	17/9	<0.001 *
Any dose change	23 (17%)	26 (58%)	<0.001 *

Initiation or termination of medications were displayed, except for loop diuretics. Any initiation/termination of medications was defined as any change. Regarding diuretics, dose-up or dose-down were displayed. Any dose-up/dose-down was defined as any dose change. The chi-square test was applied for comparison between the two groups. * *p* < 0.05.

**Table 3:** Predictors for achieving primary outcome.

	Univariable Analysis		Multivariable Analysis	
	Hazard Ratio (95% CI)	*p*-Value	Hazard Ratio (95% CI)	*p*-Value
Potential variables				
Age, years	0.99 (0.97–1.02)	0.80		
Body mass index, kg/m^2^	1.00 (0.99–1.01)	0.64		
Atrial fibrillation	1.82 (0.88–3.76)	0.10		
Left ventricular ejection fraction, %	0.98 (0.96–1.00)	0.015 *	0.99 (0.96–1.01)	0.22
Systolic blood pressure, mmHg	0.99 (0.98–1.01)	0.42		
Serum albumin, g/dL	0.36 (0.12–1.13)	0.080		
Hemoglobin, g/dL	0.94 (0.78–1.14)	0.53		
Serum creatinine, mg/dL	1.10 (0.92–1.33)	0.30		
Plasma B-type natriuretic peptide, pg/mL	1.00 (1.00–1.01)	0.14		
Use of loop diuretics	2.11 (1.02–4.34)	0.044 *	1.84 (0.83–4.06)	0.13
Open period versus blind period	0.21 (0.05–0.88)	0.033 *	0.22 (0.05–0.94)	0.041 *

CI, confidence interval. Baseline characteristics that were potentially associated with primary outcome, including the open period (versus blind period), were included in the univariable analysis to investigate potential predictors of primary outcome. Significant variables in the univariable analysis with *p* < 0.05 were included in the multivariable analysis with a forced method. The primary outcome was defined as all-cause death or heart failure admission. Day 0 was at the time of index discharge. ReDS values were blinded to the attending clinicians during the blind period, whereas ReDS values were open and diuretics were adjusted according to the ReDS values during the open period. * *p* < 0.05.

## Data Availability

Data are available upon reasonable request from the corresponding athor.
